# The influence of behavioural and socioeconomic factors on the community injury rates of adolescents assessed by the south Korean emergency medical services: an ecological approach

**DOI:** 10.1186/s12889-019-7190-6

**Published:** 2019-06-26

**Authors:** Ki Ok Ahn, Jungeun Kim, Sang Do Shin, Hyesook Park, Federico E. Vaca, Ju Ok Park

**Affiliations:** 1 Department of Emergency Medicine, Myoungji Hospital, Hanyang University College of Medicine, 55, Hwasu-ro 14beon-gil, Deogyang-gu, Goyang-si, Gyeonggi-do 10475 Republic of South Korea; 2 Laboratory of emergency medical services, Bio-medical research institute, Seoul National University Hospital, 101, Daehak-ro, Jongno-gu, Seoul, 03080 Republic of South Korea; 3 Department of Emergency Medicine, Seoul National University College of Medicine, 101, Daehak-ro, Jongno-gu, Seoul, 03080 Republic of South Korea; 4 Department of Preventive Medicine, Ewha Womans University School of Medicine, 260, Gonghang-daero, Gangseo-gu, Seoul, 07804 Republic of South Korea; 5 Department of Emergency Medicine, Yale School of Medicine, 464 Congress Avenue, Suite 260, NewHaven, CT 06519 USA; 6 Department of Emergency Medicine, Hallym University College of Medicine, Hallym University Dongtan Sacred Heart Hospital, 7, Keunjaebong-gil, Hwaseong-si, Gyeonggi-do 18450 Republic of South Korea

**Keywords:** Injury, Adolescent, Social ecology, Gender

## Abstract

**Background:**

Aim of this study is to determine if peer group risk behaviors and neighbourhood socioeconomic status (SES) would ecologically affect injury incidence according to place and gender among adolescents (aged 13–15) in South Korea.

**Methods:**

Three variables from the Korea Youth Risk Behavior Survey (2014) were used to represent peer group risk behaviours; current alcohol consumption (cAlc), the experience of violence or bullying (VicVB), and having undergone education for injury prevention (Edu-IP). The Korea Census Data (2010) was used for neighborhood SES; the degree of urbanization, the proportion of high educational attainment, and the proportion of low residential environment. The nationwide and regional Incidence-Rates of Injury assessed by EMS (IRI-EMS) were calculated according to age and gender based on the number of injuries from EMS record (2014). A linear regression model was used to examine associations.

**Results:**

The nationwide total and inside-school IRI-EMS were 623.8 and 139.3 per 100,000 population, respectively. The range of the regional IRI-EMS showed a maximum of about 4 times the difference from 345 to 1281 per 100,000 population depending on the region. The low residential environment had a significant effect on the increase of total IRI-EMS (β = 7.5, 95% CI 0.78–14.21). In the case of boys, the IRI-EMS inside-school was increased as the percentage of VicVB was higher (β = 17.0, 95% CI 1.09–32.91). In the case of girls, the IRI-EMS outside-school was increased in rural compared to urban location (β = 211.3, 95% CI 19.12–403.57).

**Conclusion:**

The incidence rate of outside-school was higher than that of inside-school, and incidence rate of boys was higher than that of girls. Peer group risk behaviors were significant only in the injury of boys. Among the SES factors, rural area was a significant factor in girls, especially outside-school injury. Moreover, the rate of households not in an apartment was significant in all outside-school injury and outside-school injury of boys.

Our study suggests that among native South Korean adolescents, neighbourhood SES and peer group risk behavior have different effects depending on the injury context such as place of occurrence or gender.

## Background

The high incidence of injuries in adolescents is a significant public health concern that requires worldwide attention. [1] The second- and third-leading causes of death worldwide in 2013 among young people aged 10–24 years were road injuries and drowning, respectively. [2] Furthermore, tens of millions of adolescents require hospital care for non-fatal injuries. According to a report on Global School-Based Student Health Surveys in 47 countries, approximately 40% of middle school students aged 13–15 years reported at least one injury in the last year. [3]

Prevention interventions for adolescent injury risk behaviours between 13 and 15 years are necessary as this age group commonly demonstrates risk behaviours associated with poor decision making and lack of experience (e.g., experimenting with alcohol use). [4] Collective peer group behaviour could serve as a major risk or protective factor in the context of adolescent injury. [5] However, many studies of adolescent injuries have handled health behaviour risk factors and injury as an outcome that was measured based on individual experience level. [6–8] To consider the strong peer influence usually prevalent in adolescents, an ecological design can evaluate the association between risks and the incidence of injury. [9]

In addition to injury risk behaviours, socioeconomic factors have been regarded as a major risk of injury among adolescents. [10–13] Socioeconomic status (SES) has a negative association with injury risk, but the level of the association varies with the type of injury, study population, and indicators of SES applied. [13] There are many studies of adolescents, and the results are inconsistent. [13, 14] The exact causality of social factors in adolescent injury is not fully understood. The current SES of youth is also important, as is the influence of the neighborhood environment (from an SES perspective) that the youth has grown up in. [15]

In previous studies, injury inside- and outside-school had different characteristics. [16] This difference may be due to different factors affecting the injury. Therefore, a contextual consideration of the location of injury in studies of adolescence is essential.

**Fig. 1:**
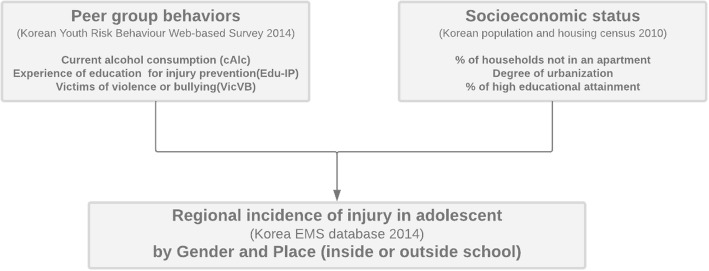
Ecological approach to the adolescent injury

This study used an ecological approach to determine if peer group risk behaviours and neighborhood SES affect injury incidence according to the place where the injury occurred and gender among adolescents in South Korea (Fig. 1).

## Methods

### Study design and setting

This cross-sectional observational study adopted an ecological approach and was conducted in the Republic of Korea (hereafter ‘Korea’). According to the 2005 Korean census, the percentage of the population between the ages of 10 and 19 was 13.9%. [17] Suicide and road traffic accidents were the first- and second-leading causes of adolescent mortality in 2015, respectively.

### Ecological effect of risk behaviours of peer group

To evaluate the ecological effect of risk behaviours of the peer group, we used data from the 2014 Korea Youth Risk Behaviour Web-based Survey (KYRBS). KYRBS was established in 2005 and is an ongoing national cross-sectional survey conducted by the Korea Centres for Disease Control and Prevention (KCDC). [18] KYRBS assesses health-risk behaviours among middle- and high-school students. KYRBS conducted a cross-sectional survey using samples that were newly extracted each year. Therefore, the temporal data for 2014 reflects the regional status at the time of the survey. Subjects to be surveyed were sampled using a multi-stage cluster sampling design nationally representing Korean middle- and high-school students aged 12–18 years. In 2014, 72,060 of 3,532,149 students were sampled. Students anonymously completed self-administered web-based questionnaires during one class period. [18] The 2014 questionnaire consisted of 125 items in 15 domains: tobacco use; alcohol use; physical activity; dietary behaviours; obesity and weight control effort; mental health; unintentional injury; oral health; personal hygiene; substance use; sexual behaviour; atopy and asthma; internet addiction; demographic variables and health equity; violence and perceived health status. [18] The variables that did not appear to be directly related to the occurrence of the injury or that did not show any statistical correlation were excluded. Finally, based on literature evidence [19] and the expert opinion of the investigators, three items were selected that likely have a direct impact on the incidence of injury: 1) current alcohol consumption (cAlc) defined as alcohol intake more than once within the last 30 days (excluding alcohol use related to ancestral rites or sacraments); 2) victims of violence or bullying (VicVB) defined as being treated by a doctor for physical violence within 12 months; and 3) having undergone formal education on injury prevention (Edu-IP) defined as safety education (about danger and preventive behaviour, first aid, and emergency evacuation and rescue) at school more than once within 12 months. The first two variables were risk behaviours, while the third was considered preventive behaviour. KYRBS variables were calculated as proportions after considering the complex survey design, selection probabilities, survey non-responses, and post-stratification using the survey procedures (proc survey data from SAS ver. 9.4 according to KCDC recommendations). Weighting factors were assigned to each KYRBS school related to the inverse probability of selection, enabling the data to represent the national population. This study used only results from students aged 13–15 years.

### Ecological effect of neighbourhood socioeconomic status

We used the 2010 Korea Census Data as an ecological index of the SES of each region. The 2010 Population and Housing Census data were collected by Statistics Korea, which is part of the Ministry of Strategy and Finance. [20] In Korea, unearned income attributable to assets such as real estate, while important, is difficult to identify, so SES cannot be evaluated using only the income level in the census. [21] Other studies have also used the residential environment and education attainment, rather than income level alone, as indicators of SES. [22] In this study, socioeconomic factors included 1) the degree of urbanisation according to administrative district standards, 2) the proportion of high educational attainment, and 3) the proportion of households not in an apartment (e.g., households in detached houses, row houses, houses within a commercial building, and others) [22] in each region. The degree of urbanisation was divided into three categories: urban, suburban, and rural. Higher educational attainment was defined as the proportion of the population with at least a high school diploma. The level of the residential environment was evaluated using the proportion of households not in an apartment. Regarding the type of housing, unlike in the United States and other western countries, most luxury houses in Korea are built as high-rise apartments. The households not in an apartment were categorised as the lower residential environment.

### The setting of regional units

We set the unit of the region that performed the analysis in this study. The National Statistical Office provides census data for 255 administrative districts in Korea. By contrast, KCDC provides KYRBS data for 46 primary survey units (PSUs) to maintain the anonymity and representativeness of the survey. To integrate the data, the Korea census reconstructed the area according to the KYRBS PSUs. The representative SES value of a PSU was defined as the average of the census values surveyed in the administrative districts belonging to each PSU, and the urbanisation level was defined using the level defined by KCDC for each PSU. After integrating these datasets, the ecological analysis was performed in 43 PSUs.

As described above, each area was subdivided into three according to the degree of urbanisation. The 43 regions included 7 rural, 21 suburban, and 15 urban regions. Urban is coded with the letter ‘U’ and a number (from U01 to U15), suburban with the letter ‘S’ and a number (from S01 to S21), and rural with the letter ‘R’ and a number (from R01 to R07).

### Outcomes

The region-specific Incidence Rates of Injury assessed by the Emergency Medical Service (IRI-EMS) were calculated. To calculate the total and gender-specific IRI-EMS, the region-specific number of injuries from the EMS records was used as the numerator and the region-specific number of registered adolescents aged 13–15 years as the denominator. In Korea, the national EMS database is maintained by EMS providers as part of their field management duties. Each provincial EMS headquarters completes the EMS run sheet, which is entered into an electronic database stored in the National Fire Agency information system. [23] The injury incidence rates were calculated for each region using the addresses of the patients recorded in the EMS database. We also used the physical address documented in the EMS for where the injury occurred. Regardless of the activity during injury and the intent to injury, if the physical address of the place of occurrence was a school, it was classified as an inside-school injury and other addresses were classified as outside-school injury. In the EMS dataset, the body part injured was described as free text by EMT and could not be used for the analysis.

### Statistical analysis

The analyses were performed using Stata ver. 15. The national IRI-EMS was calculated and the mean and standard deviation of the IRI-EMS of the 43 PSUs were calculated. The IRI-EMS was calculated as described above by region and sex. The medians and interquartile ranges of the ecological factors of the 43 PSUs were calculated and the range, minimum, and maximum were calculated to identify regional gaps. To analyze the strength and direction of linear associations, we used Pearson’s correlation coefficient. Linear regression was used to examine the association between ecological factors and IRI-EMS. The linear regression model associations were visualised using scatterplots. The analysis was performed and stratified by gender. The IRI-EMS of each region was considered a dependent variable in the multiple linear regression. In this study, all independent and dependent variables were ecological, and the unit of analysis was not an individual, but a region (i.e., a population group). In multicounty or multiregional ecological studies, multiple linear regression and Pearson correlation are used to identify significantly correlated factors. [24, 25] We tested multicollinearity by variance inflation factors (VIF) and interaction between variables. For each model, heteroskedasticity was evaluated using the Breusch-Pagan and Cook-Weisberg test (Stata command: estat hettest). A model that cannot assume homoscedasticity was adjusted to a robust standard error by the Huber / White / sandwich estimator. The level of statistical significance was set at *P* < 0.05.

## Results

**Fig. 2:**
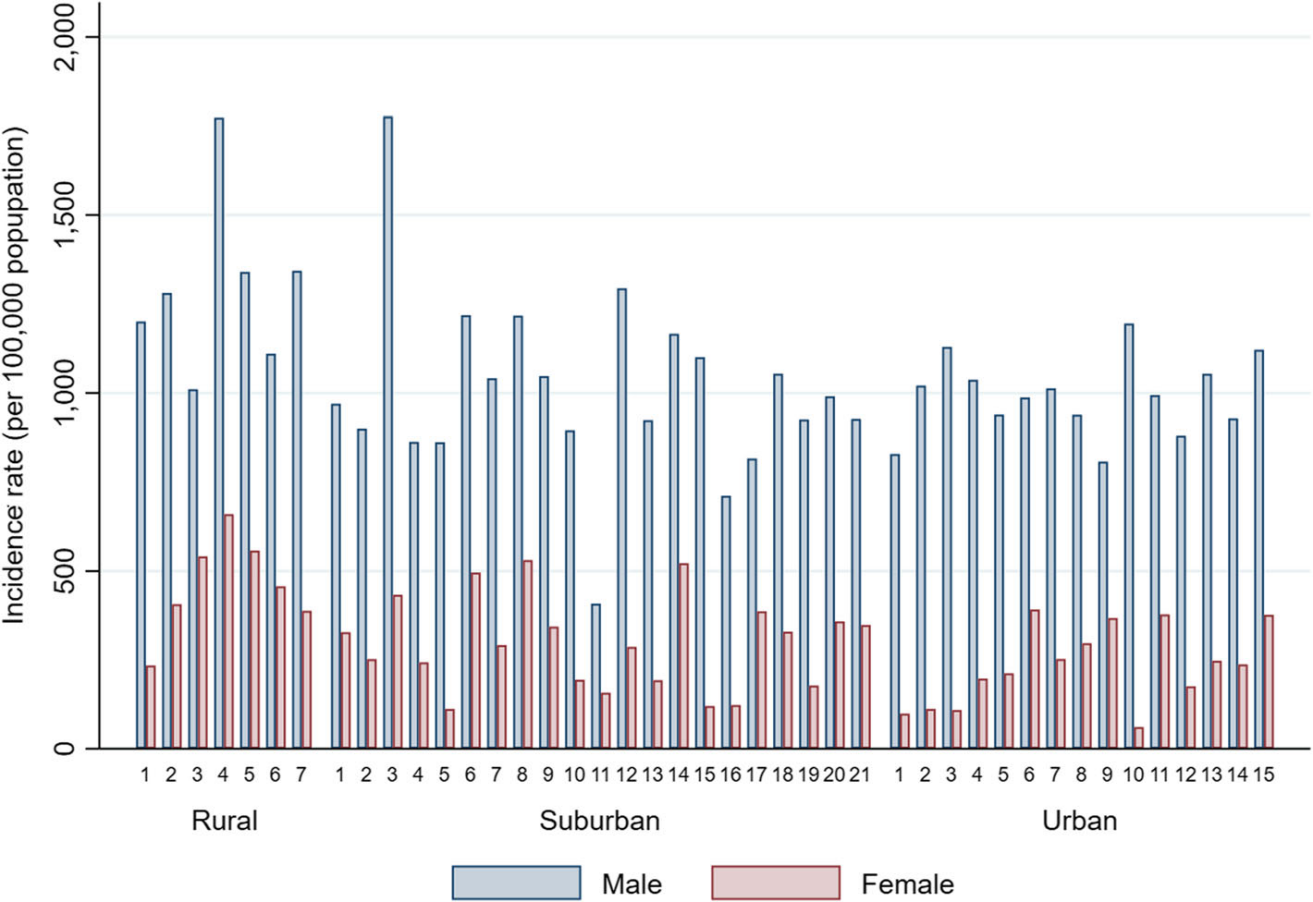
Age specific Incidence rate of injury assessed by EMS according to gender and region in Korea

In 2014, 13,154 injured patients aged 13–15 years were documented to have used the EMS. Of these, 3344 (25.4%) were girls. The nationwide IRI-EMS and IRI-EMS inside-school were 623.8 and 139.3 per 100,000 population, respectively. There was a 4-fold range in the regional IRI-EMS from 345 (region S11) to 1281 (region R04) per 100,000 population. In boys, the regional IRI-EMS ranged from 408 (region S11) to 1777 (region S03) per 100,000 population and from 60 (region U10) to 658 (region R04) per 100,000 population in girls (Fig. 2). The mean regional IRI-EMS and IRI-EMS inside-school were 750.0 (95% CI 695.9–804.2) and 156.4 (95% CI 147.2–165.6) per 100,000 population, respectively (Table 1). The median cAlc, VicVB, and Edu-IP of the 43 regions were 8.4% (IQR 7.5–9.4%), 3.1% (IQR 2.3–3.8%), and 58.2% (IQR 52.1–61.5%) for the KYRBS respondents, respectively. Edu-IP ranged from 37 to 67% depending on the region (Table 2).

**Table 1:** Mean gender- and age-specific national IRI-EMS with their 95% confidence intervals by place of injury

		IRI-EMS (per 100,000 at risk population aged 13–15)	
		Mean	95% CI
Total	All places	750.0	(695.9–804.2)
	Inside school	156.4	(147.2–165.6)
	Outside school	593.6	(543.4–643.8)
Boys	All places	1047.5	(973.8–1121.2)
	Inside school	240.3	(225.4–255.3)
	Outside school	807.2	(738.8–875.5)
Girls	All places	301.9	(257.4–346.3)
	Inside school	45.0	(38.3–51.8)
	Outside school	256.8	(216.8–296.9)

**Table 2:** Summary statistics of the regional ecological variables

		Median (IQR)	Range (Min, Max)
Peer group behaviours	cAlc (%)	8.4 (7.5,9.4)	12.0 (3.6, 15.6)
	VicVB (%)	3.1 (2.3,3.8)	4.6 (0.9, 5.5)
	Edu-IP (%)	58.2 (52.1,61.5)	29.4 (37.7, 67.1)
Socioeconomic status	High educational attainment (%)	64.6 (54.0,69.8)	42.6 (38.1, 80.7)
	Households not in an apartment (%)	7.6 (4.2,14.6)	27.5 (2.4, 29.9)

SD, standard deviation; IQR, interquartile range; Min, minimum; Max, maximum; cAlc, current alcohol consumption; VicVB, victim of violence or bullying; Edu-IP, experienced education for injury prevention

**Fig. 3:**
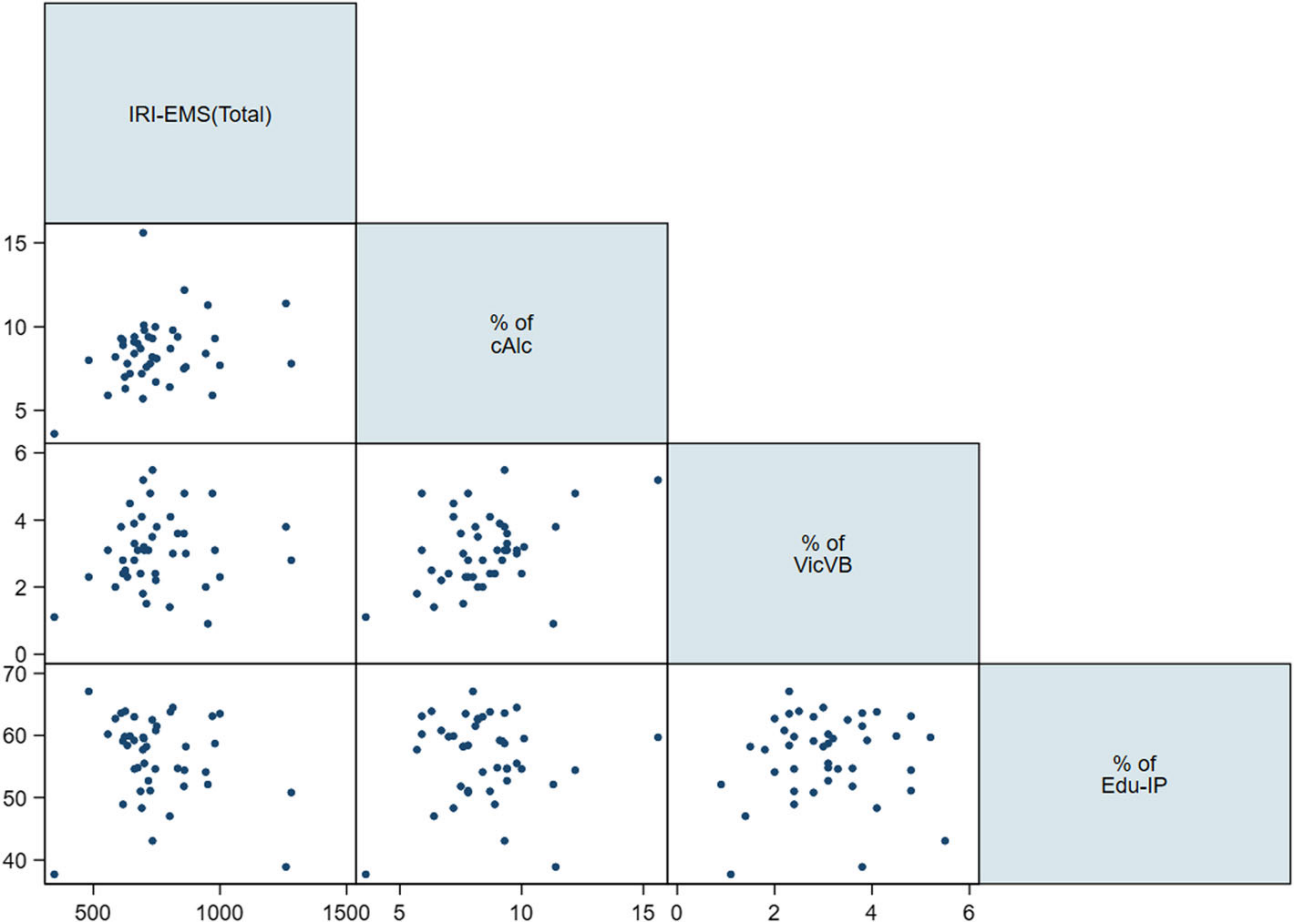
Relationship between peer group behaviors and incidence of injury

**Fig. 4:**
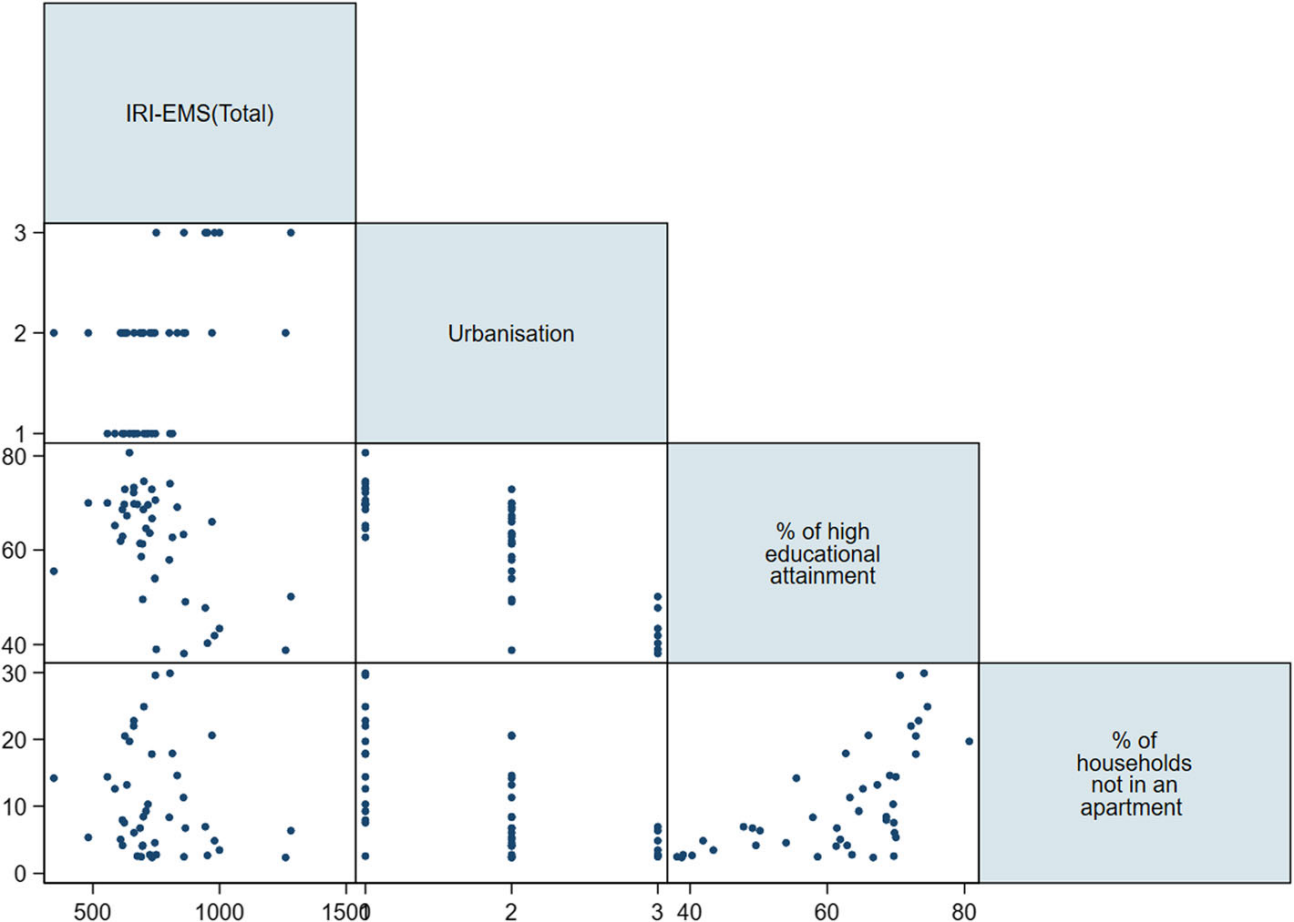
Relationship between socioeconomic status and incidence of injury

When the association between the IRI-EMS and peer group behaviours was tested using a scatterplot, cAlc showed a positive association with IRI-EMS and Edu-IP showed a negative association (Fig. 3). When the association between the total IRI-EMS and neighborhood SES was tested using a scatterplot, the degree of urbanisation showed a positive association, while the other SES variables were not significantly associated with the total IRI-EMS (Fig. 4). Pearson correlation analyses showed that cALC (r = 0.2716) and urbanization (r = 0.4872) were in moderate correlation to total IRI-EMS positively but high educational attainment (r = − 0.568) was in moderate correlation to with total IRI-EMS negatively (Table 3).

**Table 3:** Pearson’s correlation values by ecological factors and place of injury

	Peer group behaviours			Socioeconomic status		
	cAlc	VicVB	Edu-IP	Urbanization	High educational attainment	Households not in an apartment
All place	0.2716	0.1433	−0.1786	0.4872	−0.569	− 0.18
Inside-school	−0.019	0.2047	0.0816	− 0.0876	− 0.0282	0.0196
Outside-school	0.2965	0.1171	−0.2077	0.5417	−0.6087	−0.1978

cAlc, current alcohol consumption; VicVB, victim of violence or bullying; Edu-IP, experienced education for injury prevention

Table 4 shows the results of the multiple linear regression between IRI-EMS and ecological risk factors (peer group behaviours and neighborhood SES) by place of injury and gender. The proportion of households not living in an apartment had a significant effect on the increase in the total IRI-EMS regardless of the place of injury (β = 7.5, 95% confidence interval (CI) 0.78–14.21). The other factors were not significantly correlated with the total IRI-EMS. However, when analysed according to gender, the patterns differed. For boys, the IRI-EMS inside-school was significantly higher as the experience of bullying or violence was higher in the peer group (β = 17.0, 95% CI 1.09–32.91) and a lower residential environment (not in an apartment) significantly increased IRI-EMS outside-school (β = 11.0, 95% CI 1.06–21.02). For girls, the IRI-EMS outside-school was significantly increased in rural areas compared with urban areas (β = 211.3, 95% CI 19.12–403.57).

**Table 4:** Multiple linear regression between IRI-EMS and ecological risk factors (peer group behaviours and neighborhood SES) by place of injury

			All places		Inside school		Outside school	
			β (95% CI)	*p*	β (95% CI)	*p*	β (95% CI)	*p*
Total	Peer group behaviours	cAlc	8.4 (−27.52, 44.30)	0.64	−3.8 (−9.73, 2.10)	0.20	12.2 (−19.08, 43.50)	0.43
		VicVB	29.4 (− 14.48, 73.36)	0.18	9.3 (−0.56, 19.24)	0.06	20.1 (−18.77, 58.96)	0.30
		Edu-IP	−4.2 (−16.65, 8.30)	0.50	0.3 (−1.31, 2.00)	0.68	−4.5 (−15.33, 6.28)	0.40
	Neighbourhood SES	Suburban^*^	8.8 (−80.67, 98.20)	0.84	−33.5 (−77.31, 10.35)	0.13	32.5 (−45.34, 110.33)	0.40
		Rural^*^	165.1 (− 160.51, 490.72)	0.31	−42.7 (− 124.66, 39.35)	0.30	191.7 (−96.21, 479.53)	0.19
		High educational attainment	−6.9 (−18.23, 4.52)	0.23	−1.2 (−3.91, 1.60)	0.40	−6.0 (− 15.63, 3.71)	0.22
		Households not in an apartment	7.1 (−0.74, 14.90)	0.08	−0.4 (−3.19, 2.34)	0.76	7.5 (0.78, 14.21)	0.03
Boys	Peer group behaviours	cAlc	21.9 (−32.56, 76.30)	0.42	−5.0 (−14.54, 4.48)	0.29	26.9 (−18.50, 72.30)	0.24
		VicVB	40.3 (−13.97, 94.59)	0.14	17.0 (1.09, 32.91)	0.04	23.3 (−23.55, 70.15)	0.32
		Edu-IP	−10.0 (−28.53, 8.50)	0.28	1.0 (−1.64, 3.69)	0.44	−11.0 (−26.82, 4.74)	0.16
	Neighbourhood SES	Suburban^*^	−7.9 (− 129.40, 113.57)	0.90	−33.3 (− 77.08, 10.50)	0.13	25.6 (−82.16, 133.28)	0.63
		Rural^*^	254.9 (− 230.31, 740.15)	0.29	−42.9 (−125.00, 39.12)	0.30	297.5 (− 132.18, 727.22)	0.17
		High educational attainment	−4.8 (−21.86, 12.32)	0.57	−1.2 (−3.92, 1.60)	0.40	−3.6 (− 17.84, 10.61)	0.61
		Households not in an apartment	10.6 (−1.04, 22.27)	0.07	−0.4 (−3.16, 2.36)	0.77	11.0 (1.06, 21.02)	0.03
Girls	Peer group behaviours	cAlc	−1.8 (−27.14, 23.49)	0.89	−1.3 (−5.80, 3.13)	0.55	−0.5 (−22.77, 21.81)	0.97
		VicVB	9.7 (−32.63, 52.07)	0.64	2.0 (−5.44, 9.50)	0.59	7.7 (−29.61, 44.98)	0.68
		Edu-IP	−0.1 (−7.24, 6.95)	0.97	−0.6 (−1.85, 0.65)	0.34	0.4 (−5.80, 6.70)	0.89
	Neighbourhood SES	Suburban^*^	83.6 (−33.10, 200.23)	0.16	−1.3 (−21.89, 19.26)	0.90	84.9 (−17.83, 187.64)	0.10
		Rural^*^	233.5 (15.20, 451.78)	0.04	22.2 (−16.34, 60.65)	0.90	211.3 (19.12, 403.57)	0.03
		High educational attainment	−2.3 (−9.65, 5.03)	0.53	0.2 (−1.07, 1.52)	0.25	−2.5 (−9.00, 3.93)	0.43
		Households not in an apartment	5.3 (−2.08, 12.63)	0.15	0.3 (−1.04, 1.56)	0.69	5.0 (−1.46, 11.49)	0.13

CI, confidence interval; cAlc, current alcohol consumption; VicVB, victim of violence or bullying; Edu-IP, experienced education for injury prevention; SES, socioeconomic status; * referenced to urban areas

## Discussion

We observed that the socio-ecological effects of peer group risk behaviours and socioeconomic factors for injury among adolescents differed according to the location where the injury occurred and gender. The risk factors for injury differed according to the place of injury inside- and outside-school. For all adolescent injuries, SES significantly influenced the IRI-EMS outside-school. A high proportion of households not in an apartment was independently related to a high adolescent IRI-EMS outside- school. However, SES was not significantly associated with IRI-EMS inside-school. Among boys, the higher the rate of being a victim of violence in peer groups, the higher the IRI-EMS inside-school. For girls, there were no significant peer group behaviour factors for IRI-EMS. For injury inside-school, the influence of peer group behaviour factors was significant and these effects varied with gender. A poor residential neighborhood environment was associated with IRI-EMS outside-school in boys. The effect of the regional urbanisation level on IRI-EMS was significant in girls. IRI-EMS outside-school for girls in rural regions was higher than in urban and suburban regions.

Socioeconomic status is a non-modifiable risk factor, while peer group behaviour is a modifiable risk factor and is important for determining the content of injury-prevention programs. In this study, we observed that violence and bulling independently affected the IRI-EMS and these influences differed depending on the context of the injury. Therefore, behavioural changes to reduce peer group violence and bullying should be effective for inside-school injury prevention, especially in boys.

In other recent studies, the most notable school-related injuries were caused by violence and bullying. In a previous study, more than 50% of the inside-school injuries of middle school students visiting the Emergency Department were due to violence. [16] Bullying and behaviours related to violence are a serious problem and lead to a variety of health problems, including injury. [26–28] Furthermore, the victims of bullying and violence may be involved in future violence, as either victims or perpetrators. [29, 30] We observed that the rate of VicVB within the peer group was significantly related to inside-school injury in boys, but not with outside-school injury in boys or any injury in girls. Any strategy used to prevent violence and bullying in adolescents should differ by gender and age because physical violence may be more common in boys than in girls in school. [31]

Another important finding of our study was that girls in rural regions had a higher risk of outside-school injury than their counterparts in suburban and urban regions. Other studies have found higher incidence rates of fatal and non-fatal injury among rural children. [32, 33] However, it is not clear why the effects of a rural environment on injury risk differ with gender. US and Canadian studies dealing with the disparity of injury between urban and rural areas show inconsistent results for the effects of gender on injury incidence. [34–36] Further research should examine how the rural environment affects the risk of injury according to gender.

In our study, the variables used to assess neighborhood SES were obtained from the 2010 Korea census, when the cases with injury from the EMS dataset and the KYRBS respondents were elementary school students. We hypothesised that the neighborhood SES during elementary school would affect health behaviour and injury occurrence at middle school age. Caregivers, such as parents and coaches, who live in lower SES areas are more likely to have less knowledge and insufficient equipment for injury prevention at home or school. [37, 38] Children growing up in lower SES neighborhoods may also be less aware of safety and injury prevention as adolescents. Elo et al. highlight the importance of early local environments and SES conditions on later adult health outcomes. [39] Schmidt et al. supported this in a multi-level analysis based on a model in which early-life SES affected adult SES and health behaviour and reported that the effect of adolescent neighborhood SES on the occurrence of injuries in young adults varied according to gender. [40]

One strength of this study was the use of Korean nationwide population-based EMS data, which enabled the application of an ecological design. Besides national representativeness, another advantage of the EMS data was that the EMS records provided the physical address where the injury occurred. The address of the location where the injury occurred helped to gain a better understanding of the social and environmental factors affecting the occurrence of injury.

We recognize that our study had some limitations. First, IRI-EMS may have a selection bias that involves injuries of relatively high severity. In general, people with low SES and those with more severe injuries tend to use the EMS. [23] The IRI-EMS might be lower than the total incidence of injury. Therefore, our findings cannot be extrapolated to all injured patients. However, severe and significant injuries would be included. Second, the risk behaviours in the KYRBS were self-reported. Therefore, they may have been under- or overestimated due to social desirability bias, even if the KYRBS questionnaire was completed anonymously. Despite these limitations, our results have important implications for the development and application of injury-prevention policies, since this study used data that was representative at a national level. Third, the number of regional analysis units applied in this study was small, and the geographical area of each unit is large, so the neighborhood level variable may be unstable in statiscically. The KYRBS does not provide the detailed addresses of schools in each PSU to guarantee anonymity. Therefore, this study did not consider the spatial autocorrelation that might occur in SES and so linear regression was used rather than geographically weighted regression. Finally, of the three datasets used in this study, the census data were surveyed at a different time point. The Korea Census is conducted every 5 years. The closest point to the 2014 EMS dataset, which measured the outcome in this study, was the 2015 census. However, considering temporal precedence, it was a reasonable decision to apply the SES for 2010, which was measured before the outcomes were measured. We also hypothesised that the neighborhood SES during elementary school would affect health behaviour and injury occurrence at middle school age. Due to data limitations, we were unable to consider the intention and mechanism of injury in this study, so it was difficult to understand these differences fully. However, this difference may be due to the difference in injury mechanism and intention between injuries inside and outside school.

## Conclusion

The incidence of outside-school injury was higher than that of inside-school injury, and incidence was higher in boys than in girls. Each SES and peer group behavioural factor showed different effects of gender and location. Peer group violence and bullying were significant only for inside-school injuries in boys. Among the SES factors, rural area was a significant factor in girls, especially for outside-school injuries. Moreover, the proportion of households not living in an apartment was significant for all outside-school injuries and for outside-school injuries in boys, but not in girls.

As is well known, neighbourhood SES and peer group behaviour were shown to influence the community injury rate. However, our findings suggest that neighbourhood SES and peer group risk behaviour have different effects depending on the context of the injury, such as the place of occurrence (e.g., inside-school or not) and gender. These findings highlight the importance of considering the context in which an injury occurs in addition to the individual- and community-level factors that may influence the occurrence of injuries when developing injury-prevention programs for adolescents.

## Data Availability

The data that support the findings of this study are available from Korean Centers for Disease Control & Prevention (KCDC), National Fire Agency (NFA) and Statistics Korea, but restrictions apply to the availability of these data, which were used under license for the current study, and so are not publicly available. Data are however available from the authors upon reasonable request and with permission of KCDC, NFA and Statistics Korea.

## Abbreviations

cAlc: Current alcohol consumption
CI: Confidence interval
Edu-IP: education on injury prevention
IRI-EMS: Incidence Rates of Injury assessed by the Emergency Medical Service
KCDC: Korea Centres for Disease Control and Prevention
KYRBS: Korea Youth Risk Behaviour Web-based Survey
PSU: Primary survey unit
SES: Socioeconomic status
VicVB: Victims of violence or bullying
VIF: Variance inflation factors
